# Flow cytometric analysis of CD4+ T cell reactivation following anti-PD1 immunotherapy in a transgenic mouse model

**DOI:** 10.1016/j.xpro.2022.101161

**Published:** 2022-02-03

**Authors:** Lozan Sheriff, David Bending

**Affiliations:** 1 Institute of Immunology and Immunotherapy, College of Medical and Dental Sciences, University of Birmingham, Birmingham B15 2TT, UK

**Keywords:** Cell Biology, Flow Cytometry/Mass Cytometry, Cancer, Immunology, Model Organisms

## Abstract

This protocol uses the Tg4 *Nr4a3*-Tocky mouse model to recalibrate T cell activation thresholds and reveals the role that immune checkpoints play in controlling T cell activation. The example approach here uses flow cytometry to characterize quantitative and qualitative changes in splenic CD4^+^ T cells reactivated in the presence of anti-PD1 immunotherapy. The protocol is optimized for studying anti-PD1 pathway blockade only. The protocol is not compatible with cellular fixation, and T cells should be analyzed immediately after staining.

For complete details on the use and execution of this protocol, please refer to Elliot et al. (2021).

## Highlights

• We employ the Tg4 *Nr4a3-*Tocky mouse model to alter thresholds for T cell activation *in vivo*
• The protocol identifies responder T cells through re-expression of *Nr4a3*-Timer Blue
• We reveal the T cell phenotypic changes that result from anti-PD1 immunotherapy

## Graphical abstract

**Figure:**
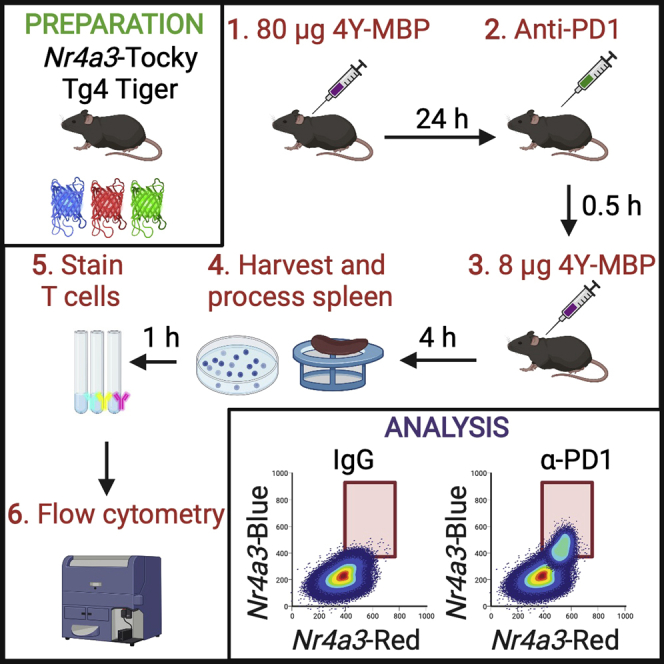

## Before you begin

Before starting with the protocol, F1 *Nr4a3*-Tocky Tg4 Tiger mouse strain should be generated. In addition, peptide, and high-quality in vivo grade blocking antibodies (shown to be functional *in vivo*) to PD1 and flow cytometry panels should all be titrated in advance. All animal experiments were approved by the local animal welfare and ethical review body and authorized under the authority of UK Home Office licenses P18A892E0A and PP3965017 (held by D.B.).

### Prepare Tg4 Tiger *Nr4a3*-Tocky mouse line

**Figure 1:**
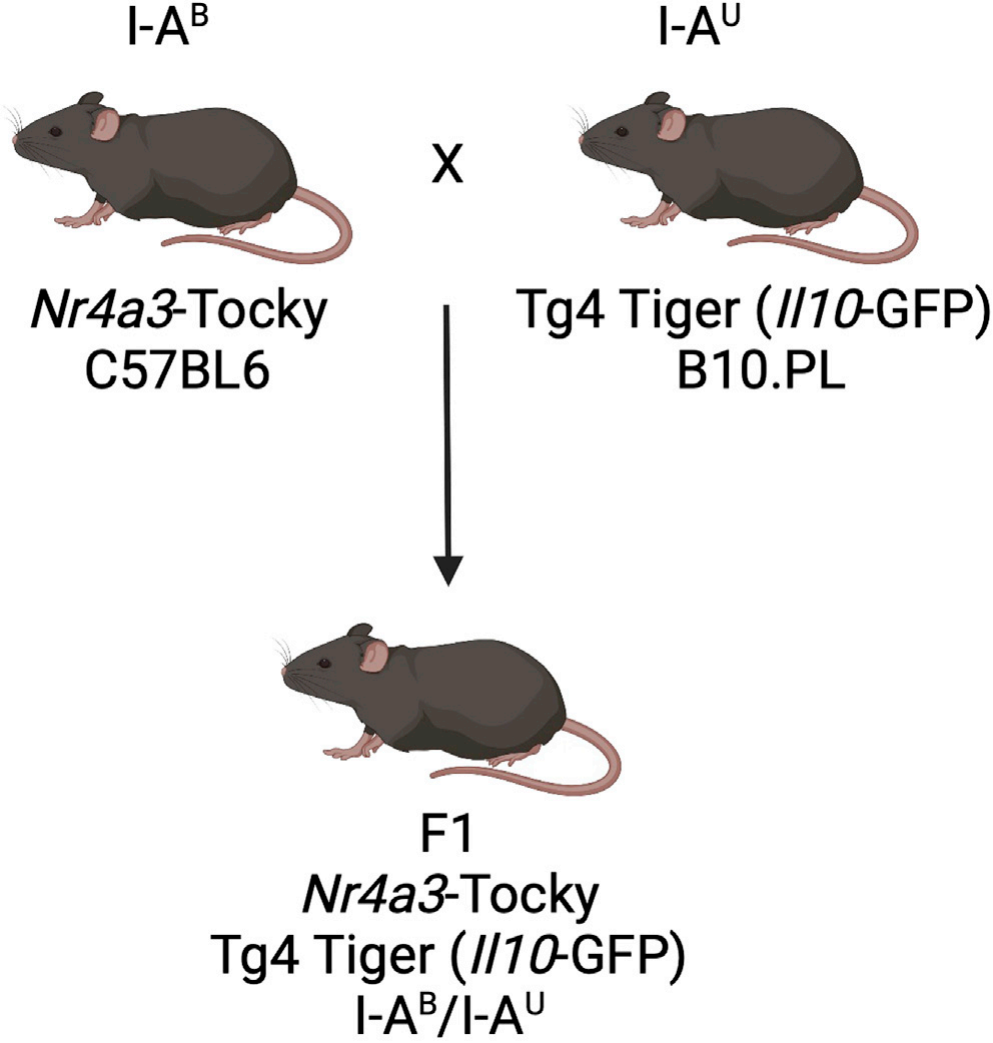
Mating strategy for generation of *Nr4a3*-Tocky Tg4 Tiger mice

The protocol uses the F1 generation from the breeding of *Nr4a3*-Tocky mice (C57BL6, I-A^b^) crossed with the Tg4 Tiger (*Il10*-GFP) line, which is on the B10.PL background (Burton et al., 2014). *Nr4a3*-Tocky mice are NFAT-responsive distal TCR signaling reporter mice that express fluorescent timer (Timer) protein (Subach et al., 2009) under the control of *Nr4a3* regulatory elements (Jennings et al., 2020). The Tg4 TCR transgenic is specific for a peptide derived from myelin basic protein (MBP) presented by the I-A^U^ MHC Class II molecule (Figure 1). We maintain colonies of *Nr4a3*-Tocky mice as homozygous BAC transgenics (Jennings et al., 2021). Similarly, Tg4 *Il10*-GFP are bred to homozygosity. This set up means no genotyping is required for the F1 generation as all will be “hemizygous” for transgenes. The protocol does not necessitate the presence of the *Il10*-GFP transgene, and Tg4 mice can be crossed with *Nr4a3*-Tocky mice for use with the protocol. The protocol depends on the expression of Timer protein to capture the re-activation of T cells within the 4 h time frame. In theory, any TCR transgenic systems could be mated to the Nr4a3-Tocky line (e.g., OTII), although these have not been experimentally tested within our laboratory.

1. Breed *Nr4a3*-Tocky homozygous mice (Jennings et al., 2020) with homozygous Tg4 *Il10*-GFP mice.
2. All mice within the F1 generation will be usable.
3. We recommend using mice at 5–6 weeks of age as, in our hands, inter-mouse experimental variance increases with age.

**CRITICAL:** Mice should be randomized for age and sex across experimental conditions. We suggest a minimum of 4–5 mice per experimental group. Troubleshooting 1

### Prepare 4Y-MBP peptide

We use an acetylated tyrosine at position 4 [4Y] variant of MBP (Ac-ASQ**Y**RPSQR). The native lysine at position 4 [4K] MBP (ASQKRPSQR) induces weak activation of T cells (Elliot et al., 2021). The [4Y] MBP variant has enhanced binding to I-A^U^ compared to the [4Y] MBP variant (Fairchild et al., 1993), making it more potent in activating T cells *in vivo*.

4. Purchase 10 mg–50 mg of MBP (custom product from GL Biochem Shanghai, sequence Ac-ASQYRPSQR, >90% purity).
5. Upon arrival, reconstitute the powder in sterile dd H_2_0 to a stock concentration of 4 mg/mL and freeze it in aliquots until use. We typically prepare 200 μL aliquots and freeze at −20°C.

**CRITICAL:** Avoid freeze/thaw cycles and always use fresh aliquots. Always keep peptides on ice when transporting.

### Prepare anti-PD1

Anti-PD1 preparation:

6. Purchase *in vivo* grade anti-PD1, clone 29F.1A12 (available from BioLegend or BioXcell) and isotype control (rat IgG2A). We use rat IgG2a (clone MAC219 (Forsyth et al., 2000)) .
7. Anti-PD1 and the isotype controls are stored in the fridge (4°C) until use.

**Table:** Suggested flow cytometer setup and panel

Antibody	Source	Clone	Dilution
Rat anti-mouse PD1 PE-Cy7	BioLegend	29F.1A12	1:200
Rat anti-mouse OX40 APC	BioLegend	OX86	1:200
Armenian hamster anti-human/mouse ICOS AF700	BioLegend	C398.4A	1:50
eFluor-780 fixable viability dye	eBioscience	NA	1:2000
Rat anti-mouse CD4 BUV737	BD Biosciences	GK1.5	1:200
Mouse anti-mouse TCR Vbeta8.1,8.2 BUV395	BD Biosciences	MR5-2	1:200

The panel design has been optimized to minimize interference with the Timer-Blue channel (excitation 405 nm, detection 450/50 nm filters). We have previously shown that at physiological expression levels, GFP (excitation 488 nm detection 530/30 nm filers), Timer Blue and Timer Red (excitation 561 nm, detection 610/20 nm filters) show negligible compensation requirements (Jennings et al., 2021). A key compensation consideration is the overlap of PE-Cy7 and PE-Texas Red (Timer-Red) channels. We strongly recommend that for PE-Cy7 compensation, the same antibody is used for single color controls. Troubleshooting 2

**Table:** Example BD LSR Fortessa X20 cytometer set up

Detector name	Laser	Filters (nm)	Fluorochrome	Voltage (volts)
**FITC**	488 nm	530/30	GFP	403
**BV421**	405 nm	540/50	Timer Blue	412
**PE-Texas Red**	561 nm	610/20	Timer Red	610
**PE-Cy7**	561 nm	795/71	PE-Cy7	521
**APC**	640 nm	670/30	APC	580
**APC-Alexa 700**	640 nm	730/45	AF700	530
**APC-Cy7**	640 nm	780/60	eFluor 780	481
**BUV395**	355 nm	379/28	BUV395	559
**BUV7377**	355 nm	740/35	BUV737	700

**Table:** Example compensation matrix (% overlap)

	GFP (FITC)	Blue (BV421)	Red (PE-Texas Red)	PE-Cy7	APC	AF700	APC-Cy7	BUV395	BUV737
**GFP (FITC)**	100	0	0	0	0	0	0	0	0
**Blue (BV421)**	0	100	0	0	0	0	0	0	0
**Red (PE-Texas Red)**	0	0	100	2	0	0	0	0	0
**PE-Cy7**	0.1	0	2.6	100	0	0.4	3.7	0	3.3
**APC**	0	0	0.2	1.7	100	32.6	3.9	0	0
**AF700**	0	0	0	3.3	3.7	100	11.5	0	12
**APC-Cy7**	0.7	0	0.4	23	3.2	12	100	0	13.5
**BUV395**	0	0.2	0	0	0	0	0	100	0
**BUV737**	0	0	0	0.2	0.1	8.3	1.8	0.9	100

For further details regarding compensation matrix considerations and fluorochrome compatibility with *Nr4a3*-Timer Red, *Nr4a3*-Timer Blue and GFP please refer to (Jennings et al., 2021). Troubleshooting 3

## Key resources table

**Table:** 

REAGENT or RESOURCE	SOURCE	IDENTIFIER
**Antibodies**		

Rat anti-mouse CD4 BUV737 (clone GK1.5, 1 in 200 dilution)	BD Biosciences	Cat# 612761; RRID: AB_2870092
Mouse anti-mouse TCR Vb8.1,8.2 BUV395 (clone MR5-2, 1 in 200 dilution)	BD Biosciences	Cat# 744335; RRID: AB_2742163
Armenian hamster anti-human/mouse ICOS AF700 (clone C398.4A, 1 in 50 dilution)	BioLegend	Cat# 313528; RRID: AB_2566126
Rat anti-mouse PD1 PE-Cy7 (clone 29F.1A12, 1 in 200 dilution)	BioLegend	Cat# 135215; RRID: AB_10696422
Rat anti-mouse OX40 APC (clone OX-86, 1 in 200 dilution)	BioLegend	Cat# 119413; RRID: AB_2561723
GoInVivo Purified anti-mouse PD-1 (clone 29F.1A12)	BioLegend	Cat# 135233; RRID: AB_2616834
InVivo Mab rat anti-mouse PD-1 (clone 29F.1A12) (alternative supplier)	Bio X Cell	Cat# BE0273; RRID: AB_2687796
Rat IgG2a (clone MAC219)	Kind gift from Prof Anne Cooke (University of Cambridge)	PMID: 10810307

**Chemicals, peptides, and recombinant proteins**		

MBP Ac1-9[4Y] peptide AcASQYRPSQR	GL Biochem Shanghai	Custom product
Phosphate buffered saline (Ca^2+^ Mg^2+^ free)	Thermo Fisher Scientific	Cat# 14190-094

**Critical commercial assays**		

eFluor-780 fixable viability dye (1 in 2000)	eBioscience	Cat# 65-0865-14
eBioscience 1X RBC lysis buffer	Thermo Fisher Scientific	Cat# 00-4333-57

**Experimental models: Organisms/strains**		

*Nr4a3*-Tocky	Bending et al. (2018)	PMID: 29941474
Tg4 *Il10-*GFP “Tiger”	Burton et al. (2014)	PMID: 25182274

**Software and algorithms**		

GraphPad Prism 9	GraphPad Inc	https://www.graphpad.com/scientific-software/prism/
FlowJo v10	BD Biosciences	https://www.flowjo.com/solutions/flowjo

**Other**		

BD LSR Fortessa X20	BD Biosciences	Custom product

## Step-by-step method details

### Primary immunization

**Timing: 24 h**

1. Primary immunization of mice with 80 μg [4Y] MBP
  a. Prepare [4Y] MBP at a concentration of 0.4 mg/ mL in 1× PBS, with 200 μL volume required per mouse, plus 10% extra as buffer. Keep on ice.
  b. Inject Tg4 Tiger *Nr4a3*-Tocky mice with 200 μL peptide (80 μg total dose) subcutaneously into the right flank using a 0.5 mL insulin needle and syringe.
  c. After injection of the peptide, remove the needle and gently pinch skin for 10 s to minimize peptide/ PBS leakage.
  d. Leave mice for 24 h.

**CRITICAL:** A bolus shape should form under the skin if injection is subcutaneous. If significant amounts of peptide/PBS solution leaks after injection (>5–10 μL) then that mouse should be excluded from the experiment.

### Administration of anti-PD1 immunotherapy

**Timing: 30–60 min**

2. Preparation and injection of anti-PD1 antibodies
  a. Prepare anti-PD1 and isotype control antibodies at a concentration of 2.5 mg/ mL in sterile 1× PBS. You will require 200 μL per mouse plus 10% excess volume.
  b. Inject 0.5 mg (200 μL) of anti-PD1 or isotype control i.p. 24 h after the primary immunization.
  c. Wait for 30 min.

**CRITICAL:** It is crucial to allow a minimum of 30 minutes for the antibodies to distribute in the mouse before re-challenge with peptide. Troubleshooting 4

### Peptide re-challenge of mice

**Timing: 4 h**

3. Rechallenge of mice with 8 μg [4Y] MBP
  a. Prepare [4Y] MBP at a concentration of 0.04 mg/ mL in 1× PBS, with 200 μL volume required per mouse, plus 10% extra as buffer. Keep on ice.
  b. Inject Tg4 Tiger *Nr4a3*-Tocky mice with 200 μL peptide (8 μg total dose) subcutaneously into the left flank using a 0.5 mL insulin needle and syringe.
  c. After injection of the peptide, remove the needle and gently pinch skin to for 10 s to minimize peptide/ PBS leakage.
  d. Leave mice for 4 h.

***Note:*** 8 μg is a recommended rechallenge dose. However, lowering or raising this dose will alter the proportion of responder T cells (see Figure 4 (Elliot et al., 2021))

**CRITICAL:** A bolus shape should form under the skin if injection is subcutaneous. If significant amounts of peptide/PBS solution leaks after injection (>5–10 μL) then that mouse should be excluded from the experiment.

### Spleen harvest and generation of splenocyte suspensions

**Timing: 1–2 h**

4. After 4 h peptide rechallenge, euthanize mice, and process spleen for flow cytometric staining
  a. Mice are euthanized and the whole spleen is removed intact from the Tg4 *Nr4a3*-Tocky mice. Ensure complete detachment of the pancreas and place it into a 1.5 mL Eppendorf tube containing 0.5 mL of 2% FBS/ PBS (v/v).
  b. Into a sterile 5 mL petri dish, place a 70 μm cell strainer and add the spleen and 1 mL of 2% FBS PBS buffer. (Alternatively, a strainer can be placed in a 50 mL Falcon tube for processing).
  c. Using the syringe plunger from a 5 mL syringe, gently force the spleen through the cell strainer.
  d. Using a P1000 pipette add 1 mL 2% FBS PBS buffer to wash the strainer.
  e. Filter the splenocyte solution back through the strainer by taking from suspension in the petri dish and pipetting back onto the strainer.
    i) Repeat this 8–10 times using P1000 pipette until suspension is homogenous.
  f. Transfer the splenocyte suspension from each mouse into individual 15 mL Falcon tubes.
  g. Wash strainer with 1 mL 2 % FBS PBS and transfer this to the 15 mL Falcon tube.
  h. Centrifuge Falcon tube at 500 *g* for 5 min at 15°C–25°C.
  i. Decant supernatant and proceed to perform red blood cell lysis by resuspending the cell pellet in 1 mL of RBC lysis buffer.
  j. Incubate for 2 min on ice.
  k. Top up falcon tube to 10 mL with cold 2% FBS PBS and centrifuge at 500 *g* for 5 min at 4°C.
  l. Decant supernatant and resuspend splenocytes in 2 mL ice cold 2% FBS PBS.
    i. Remove with a P1000 pipette any cell clumps that may have formed.
  m. Transfer 100 μL of splenocyte suspension (typically 2–5M splenocytes) to a 96 well U bottom plate for staining. Alternatively, cells can be stained in 5 mL FACS tubes.

### Flow cytometric staining

**Timing: 1 h**

5. Flow cytometric staining (for 96 well plate staining)
  a. Centrifuge 96 well plate at 500 *g* for 3 min at 15°C–25°C.
  b. Prepare master mix of antibodies in 2% FBS PBS containing 1 in 2000 dilution of fixable viability dye eFluor780, as per table below.
    ***Optional:*** For splenocyte analysis we do not routinely perform Fc receptor block, however this can be included at this stage.

**Table:** 

Antibody	Dilution	Volume to add to 1 mL
PD1 PE-Cy7	1:200	5 μL
OX40 APC	1:200	5 μL
ICOS AF700	1:50	20 μL
CD4 BUV737	1:200	5 μL
TCR Vbeta8.1,8.2 BUV395	1:200	5 μL
  c. Stain samples in 25 μL of antibody master mix.
    **CRITICAL:** Remember to include compensation controls. Unstained splenocytes can be used for compensation of *Nr4a3*-Timer Red (PE-Texas Red channel) as per (Jennings et al., 2021). As stated previously, the staining panel design is optimized to not require compensation between *Nr4a3*-Timer Blue (BV421 channel), GFP (FITC channel) and the other channels. For tandem dyes like PE-Cy7 use the same antibody from the panel master mix. Typical cytometer settings, channels and compensation matrix are shown earlier for reference, suggested controls are listed below. NB Timer Blue and Timer Red are excited by different lasers (405 nm versus 561 nm) and emit in different ranges (Timer Blue ∼ 450 nm, Timer Red ∼ 610 nm) so compensation between these two channels is not necessary. Troubleshooting 2

**Table:** 

Channel name	Panel stain	Compensation control suggestion
**FITC**	*Il10-*GFP	GFP expressing splenocytes/comp beads
**BV421**	*Nr4a3*-Timer Blue	Not usually required if following suggested panel design, but can use 4 h anti-CD3 stimulated *Nr4a3*-Tocky splenocytes
**PE-Texas Red**	*Nr4a3*-Timer Red	*Nr4a3-*Tocky splenocytes
**PE-Cy7**	PD1 PE-Cy7	PD1 PE-Cy7
**APC**	OX40 APC	Any APC antibody
**APC-Alexa 700**	ICOS AF700	Any AF700 antibody
**APC-Cy7**	eFluor 780 viability dye	eFluor 780 viability dye
**BUV395**	TCRvBeta8.1/8.2 BUV395	TCRvBetab8.1/8.2 BUV395
**BUV7377**	CD4 BUV737	CD4 BUV737
  d. Incubate plate at 4°C for 20 min.
  e. Add 180 μL of ice cold 2% FBS PBS to each well and centrifuge plate at 500 *g* for 3 min at 15°C–25°C.
  f. Decant supernatant and resuspend pellets in 150 μL 2% FBS PBS and transfer to 5 mL FACS tubes.
  g. Wells can be rinsed with 150 μL 2% FBS PBS and combined in FACS tube to give a total volume of 300 μL for acquisition.
  h. Keep cells on ice in dark and acquire data on flow cytometer within 2–4 h.
    **CRITICAL:** Do not fix cells as this will result in loss of *Nr4a3*-Timer Blue fluorescence. Due to slow maturation of Timer protein from Blue to Red it is advised that data are acquired soon after completion of staining. Troubleshooting 5

## Expected outcomes

**Figure 2:**
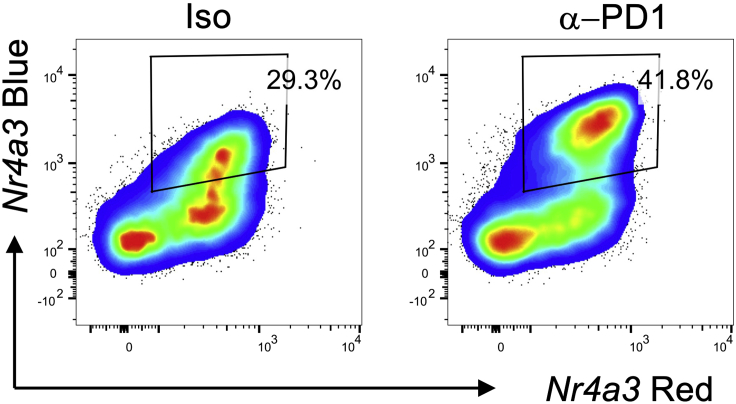
Increased proportion of responders in response to anti-PD1 treatment Tg4 *Nr4a3*-Tocky *Il10*-GFP mice were immunized s.c. with 80 μg of [4Y] MBP. 24 h later mice were randomized to receive 0.5 mg isotype or 0.5 mg anti-PD1 30 min prior to re-challenge with 8 μg [4Y] MBP peptide. Cells are gated on live CD4^+^Vβ8.1/8.2^+^ T cells and analyzed for expression of *Nr4a3*-Blue vs. *Nr4a3*-Red 4 h after peptide rechallenge. Gates are set on the responder T cell population.

**Figure 3:**
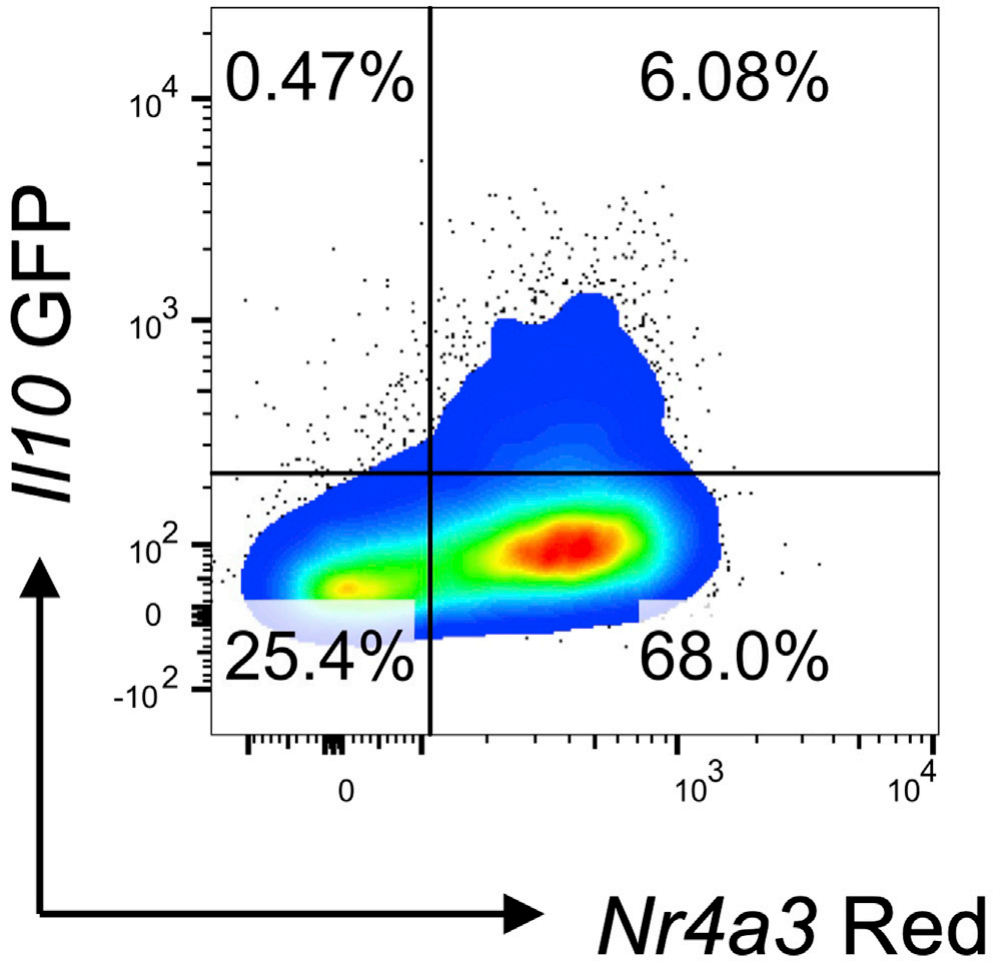
Example of *Il10*-GFP expression Tg4 *Nr4a3*-Tocky *Il10*-GFP mice were immunized s.c. with 80 μg of [4Y] MBP. 24 h later the mouse received 0.5 mg isotype prior to re-challenge with 8 μg [4Y] MBP peptide. Cells are gated on live CD4^+^Vβ8.1/8.2^+^ T cells and analyzed for expression of *Nr4a3*-Red vs. *Il10*-GFP 4 h after peptide rechallenge.

Treatment with anti-PD1 will increase the proportion of T cells that re-activate in response to re-challenge with the 8-μg dose of peptide in comparison to isotype treated mice. These cells are defined as the percentage of CD4^+^ T cells that are *Nr4a3*-Timer Blue^+^Red^+^ (Figure 2). Furthermore, analysis of CD4^+^ *Nr4a3*-Timer Blue^+^Red^+^ T cells will reveal that the median level of *Nr4a3*-Timer Blue and OX40 will be higher in responder T cells in the anti-PD1 treated group compared to isotype treated mice (Elliot et al., 2021). In addition, *Il10*-GFP expression will be found within the *Nr4a3*-Timer Red fraction (Figure 3).

## Quantification and statistical analysis

**Figure 4:**
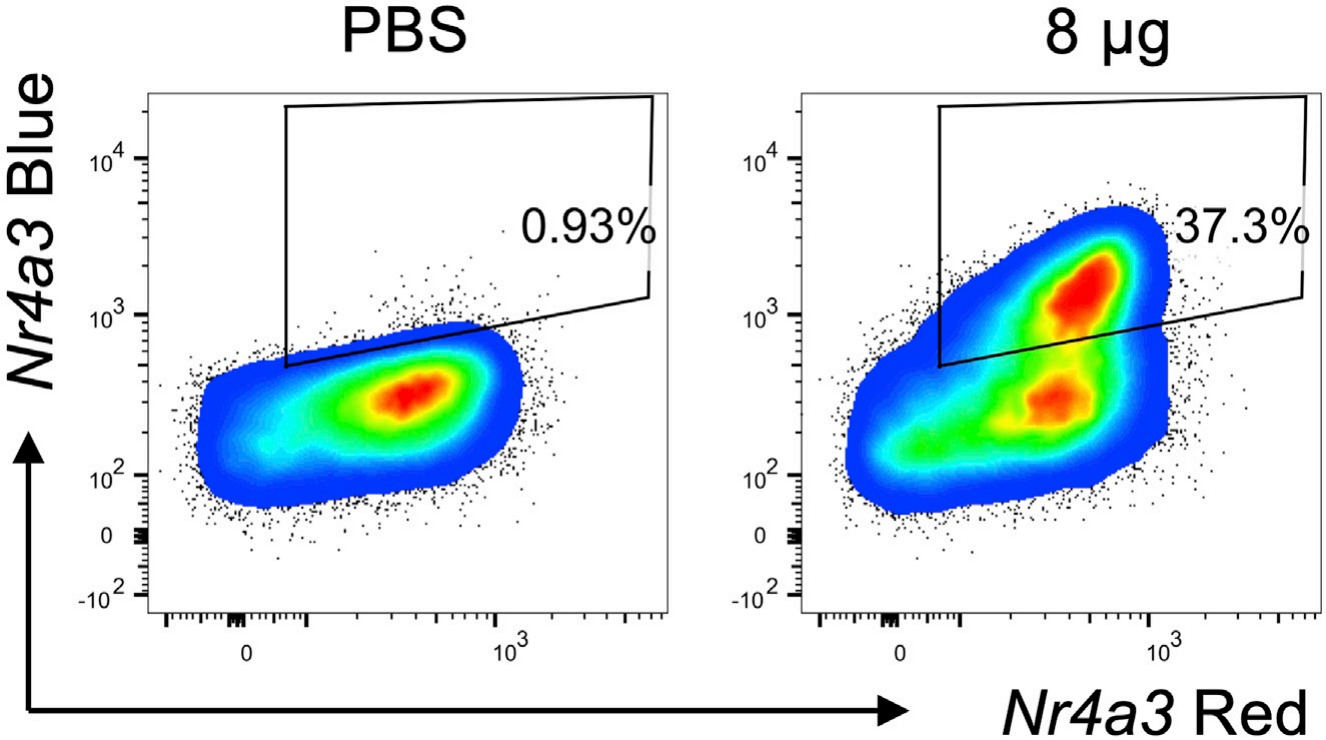
Gating strategy for identifying “responder” T cells Tg4 *Nr4a3*-Tocky *Il10*-GFP mice were immunized s.c. with 80 μg of [4Y] MBP. 24 h later mice received either PBS or 8 μg [4Y] MBP peptide. Cells are gated on live CD4^+^Vβ8.1/8.2^+^ T cells and analyzed for expression of *Nr4a3*-Blue vs. *Nr4a3*-Red 4 h after peptide rechallenge. Gates are set on the responder T cell population.

Flow cytometry gates to determine responder populations can be set by including a mouse that receives the primary immunization (80 μg [4Y] MBP) but only PBS on the secondary restimulation. Most T cells, after 24 h immunization with 80 μg [4Y] MBP, are *Nr4a3*-Timer Red^+^Blue^-^ (Figure 4). When a second immunization with 8 μg [4Y] MBP is performed a clear Blue^+^Red^+^ population is observable. These are the “responder” cells. Gating on this population will allow a comparison of the phenotype of responder cells between treatment groups.

**Figure 5:**
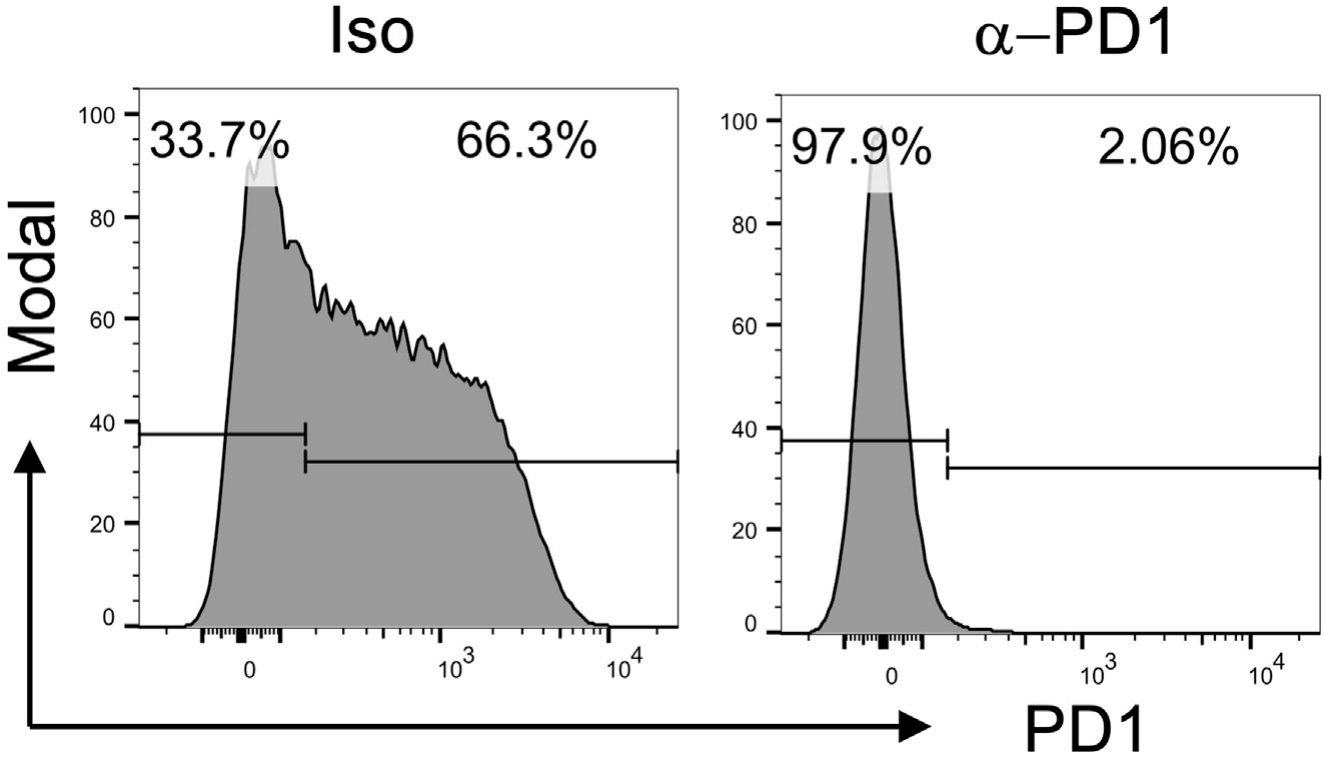
Confirmation of successful PD1 blockade *in vivo* Tg4 *Nr4a3*-Tocky *Il10*-GFP mice were immunized s.c. with 80 μg of [4Y] MBP. 24 h later mice were randomized to receive 0.5 mg isotype or 0.5 mg anti-PD1 30 min prior to re-challenge with 8 μg [4Y] MBP peptide. Cells are gated on live CD4^+^Vβ8.1/8.2^+^ T cells and then analyzed for expression of PD1 4 h after peptide rechallenge.

As part of a quality control step, we include a fluorochrome conjugated anti-PD1 antibody of the same clone as the blocking antibody administered *in vivo*. This allows confirmation of successful i.p. injection and that PD1 is blocked (Figure 5). Where significant PD1 staining remains, this indicates inadequate blocking of PD1, and such mice should be excluded from further analysis.

## Limitations

The protocol here is optimized for anti-PD1 immunotherapy but has been adapted for other immune checkpoints such as Lag3; however, for other receptors confirmation that the receptor and its ligand are expressed in the splenic environment would be required. Due to the mixed mouse background, generation of gene knockouts is not straightforward, and would require backcrossing onto the B10.PL background.

The protocol is specific for CD4^+^ T cells, but theoretically could be adapted to CD8^+^ T cells using a CD8^+^ TCR transgenic. However, for all analyses of responder T cells, the protocol is not compatible with fixation and intranuclear staining and samples need to be analyzed soon after completion of flow cytometric staining.

## Troubleshooting

### Problem 1

High variability between mice (steps 1–3).

### Potential solution

High variability is often driven by inadequate randomization. We typically use mice 5–6 weeks of age with weights 18–24 g. We ensure mice are randomized based on sex and age, as both influence weight. As multiple injections are given over time, large variance in mice weight can give rise to wider ranges in responses. One way to circumvent this is to administer doses on a mg/ kg basis. We base our 80-μg dose on an approximate mouse weight of 20 g. This equates to doses of 4 mg/kg for primary immunization and 0.4 mg/kg for re-challenge. For antibody, 0.5 mg would equate to 25 mg/kg.

### Problem 2

Timer-Red channel shows a skewed distribution (step 5).

### Potential solution

This is typically a result of over or under compensation of PE-Cy7 and Timer Red (PE-Texas Red channel). In our experience PE-Cy7 tandem dyes exhibit subtle differences in compensation between batches and brands. It is imperative to use the same PE-Cy7 antibody as in the panel (either using beads compensation, or staining of cells expressing the ligand).

### Problem 3

Timer-Blue and Timer-Red channels show diagonal autofluorescence (step 5).

### Potential solution

You can utilize a “dump” channel to remove noise. We recommend using the PerCP-Cy5.5 channel (excited by a 488 nm laser, with 710/50 nm filters) to remove cells showing signal in this channel.

### Problem 4

Minimal effect of anti-PD1 on T cell reactivation (step 2c).

### Potential solution

There are several potential reasons for this. (1) A key part of the protocol is the minimum 30-min gap between administration of the anti-PD1 antibody and re-challenge with [4Y] MBP. This gap can be extended to increase the time for maximal blockade of the pathway for antibodies with potential different distribution half-lives. (2) It is strongly recommended that receptor blockade is confirmed through counter staining for the marker of interest. In our hands, a non-response is almost always due to a failed i.p. injection as evidenced by high PD1 staining.

### Problem 5

Minimal Timer-Blue detected (steps 3–5).

### Potential solution

This could represent a failed subcutaneous injection of 8 ug [4Y] MBP, which would result in no restimulation of the T cells *in vivo*. Conversely, this can arise if cells are left overnight in the fridge or fixed, as Timer Blue will undergo slow maturation to the terminal Red form, resulting in loss of Blue signal. It is imperative that the experiment is planned so that flow cytometric analysis can occur soon after completion of staining.

## Resource availability

### Lead contact

Further information and requests for resources and reagents should be directed to and will be fulfilled by the lead contact, Dr David Bending, d.a.bending@bham.ac.uk .

### Materials availability

*Nr4a3*-Tocky mice are available under MTA from Dr Masahiro Ono (Imperial College London, UK).

## Data Availability

Data underlying this protocol are available from the lead contact upon reasonable request.
